# Health services changes: is a run-in period necessary before evaluation in randomised clinical trials?

**DOI:** 10.1186/1745-6215-15-41

**Published:** 2014-01-30

**Authors:** Trishna Rathod, John Belcher, Alan A Montgomery, Chris Salisbury, Nadine E Foster

**Affiliations:** 1 Research Institute for Primary Care & Health Sciences, Keele University, Keele, Staffordshire ST5 5BG, UK; 2 School of Computing and Mathematics, Keele University, Keele, Staffordshire ST5 5BG, UK; 3 Nottingham Clinical Trials Unit, University of Nottingham, Queen’s Medical Centre, Nottingham NG7 2UH, UK; 4 Centre for Academic Primary Care, School of Community and Social Medicine, University of Bristol, Canynge Hall, 39 Whatley Road, Bristol BS8 2PS, UK

**Keywords:** Health services, Learning curve, Randomised clinical trial, Run-in period

## Abstract

**Background:**

Most randomised clinical trials (RCTs) testing a new health service do not allow a run-in period of consolidation before evaluating the new approach. Consequently, health professionals involved may feel insufficiently familiar or confident, or that new processes or systems that are integral to the service are insufficiently embedded in routine care prior to definitive evaluation in a RCT. This study aimed to determine the optimal run-in period for a new physiotherapy-led telephone assessment and treatment service known as PhysioDirect and whether a run-in was needed prior to evaluating outcomes in an RCT.

**Methods:**

The PhysioDirect trial assessed whether PhysioDirect was as effective as usual care. Prior to the main trial, a run-in of up to 12 weeks was permitted to facilitate physiotherapists to become confident in delivering the new service. Outcomes collected from the run-in and main trial were length of telephone calls within the PhysioDirect service and patients’ physical function (SF-36v2 questionnaire) and Measure Yourself Medical Outcome Profile v2 collected at baseline and six months. Joinpoint regression determined how long it had taken call times to stabilise. Analysis of covariance determined whether patients’ physical function at six months changed from the run-in to the main trial.

**Results:**

Mean PhysioDirect call times (minutes) were higher in the run-in (31 (SD: 12.6)) than in the main trial (25 (SD: 11.6)). Each physiotherapist needed to answer 42 (95% CI: 20,56) calls for their mean call time to stabilise at 25 minutes per call; this took a minimum of seven weeks. For patients’ physical function, PhysioDirect was equally clinically effective as usual care during both the run-in (0.17 (95% CI: -0.91,1.24)) and main trial (-0.01 (95% CI: -0.80,0.79)).

**Conclusions:**

A run-in was not needed in a large trial testing PhysioDirect services in terms of patient outcomes. A learning curve was evident in the process measure of telephone call length. This decreased during the run-in and stabilised prior to commencement of the main trial. Future trials should build in a run-in if it is anticipated that learning would have an effect on patient outcome.

**Trial registration:**

Current Controlled Trials, ISRCTN55666618

## Background

In randomised clinical trials (RCTs) within health services research, it is common that new or amended services are developed, implemented and evaluated almost immediately. This may not be ideal as new services may benefit from amendments based on early learning and the service might demonstrate improved performance over time [1], as new processes and practices become embedded, known as a learning curve. Consequently, an immediate evaluation of the new service as part of an RCT may be misleading and subject to bias.

If it is known or suspected that a learning curve may affect a new service or treatment approach, a run-in period used as a period of consolidation is suggested prior to recruitment to the RCT [2]. Such a run-in period allows healthcare practitioners to become sufficiently familiar in delivery of the new service and for new processes to become embedded in routine care, thus allowing the service to reach a stable and efficient level of delivery before evaluation. By way of example, a cardiopulmonary resuscitation study had a three month run-in period for practitioners to become familiar in using a new monitor defibrillator prior to the start of the formal RCT evaluation [3].

Although run-in periods are used for a variety of reasons [4-6] using them as a period of consolidation appears to be relatively rare in health services research as they can require considerable time and resource prior to a full RCT. As a result, clinical research teams may be hesitant to incorporate run-in periods in the timeline of their RCTs. Very little is known about whether run-in periods of consolidation are necessary prior to RCTs of new services or treatment approaches or whether they have an impact on either key intervention processes or clinical outcomes.

The aims of this study were:

1. To determine the optimal run-in period of consolidation for a new primary care service prior to the main RCT.

2. To determine if the provision of a run-in period of consolidation was needed before patient and process outcomes were evaluated.

## Methods

### PhysioDirect trial

Musculoskeletal problems are painful and disabling and can cause poor health; such individuals are often referred to physiotherapy for treatment [7]. Access to physiotherapy in the UK NHS has traditionally been associated with long waiting times for an initial consultation, with delays of up to several months [7]. Consequently, many patients fail to attend their consultation or the consultation provides little benefit as patients are seen too late [8]. In response to waiting list pressures, new physiotherapy-led telephone assessment and treatment services known as PhysioDirect have been developed and are available in some areas of the UK [8]. The MRC PhysioDirect trial was a multi-centre pragmatic equivalence RCT designed to test the clinical and cost-effectiveness of PhysioDirect services. Patients were randomised in a 2:1 ratio to PhysioDirect or usual physiotherapy care where patients were placed onto waiting lists and waited for the next available appointment [9]. The trial incorporated a run-in period of consolidation of between seven and twelve weeks varying between the participating primary care trusts (PCTs) prior to the main trial. The data from this trial will be used for the purposes of this paper.

Multisite research ethics approval for the PhysioDirect trial was obtained from Southmead research ethics committee, reference 08/H0102/95. All participants gave informed written consent.

In the PhysioDirect trial, in the PhysioDirect service, patients wishing to access physiotherapy treatment could telephone a senior physiotherapist for an initial assessment followed by appropriate advice over the telephone supplemented by leaflets through the post. Physiotherapists used bespoke software to provide a template for their assessment and as an electronic medical record of the consultation. Patients were invited to telephone the service two to four weeks later to report progress. Patients were invited to a face to face consultation if they needed urgent care or if their condition failed to improve.

A new PhysioDirect service was set up in four PCTs. As PhysioDirect was a new service, eight physiotherapists from each PCT (32 in total) undertook a structured training programme and had to be certified as competent to deliver the service, however it was expected the physiotherapists would require a period of practice and consolidation before they became familiar with delivering the new service. To ensure the PhysioDirect service was running efficiently prior to its formal evaluation in the main RCT, the PhysioDirect trial operated in two phases. The first phase was a run-in period of consolidation which lasted 12, 10, 11 and 7 weeks for PCTs A, B, C and D respectively. Patients recruited during this phase were randomised to either the usual care arm or to the PhysioDirect arm. The second phase was the main trial which recruited and randomised a fresh set of patients to the two arms. Patients randomised in the run-in period were not included in the main trial analysis, thus creating two separate datasets for analysis in this study. The run-in period allowed physiotherapists to build their confidence and competence in delivering the PhysioDirect service and to embed key processes. In particular, it was anticipated that the length of telephone calls would decrease as the physiotherapists gained confidence in consulting with patients by telephone and in using the PhysioDirect software.

By comparing the data collected from the run-in period with those from the main trial, we examined two issues. First, whether the length of telephone consultations changed over time, as this could have important implications for the cost-effectiveness of the PhysioDirect service. Second, whether patients’ clinical outcomes differed between those who were recruited in the run-in period and main trial.

### Outcomes

Key outcomes for this study include both process and clinical outcomes. The key process outcome was the length of the telephone call in the PhysioDirect service. The date and call time of each telephone call made by a patient to the PhysioDirect service and the physiotherapist who handled the call was recorded. Only the participant’s initial call was used in the analysis as follow up call times were shorter as patient details were already known.

Two key clinical outcomes were used for the purposes of this study. The first was the primary clinical outcome for the trial, the physical component score (PCS), from the SF-36v2 patient reported questionnaire of general health. PCS is a generic measure of physical function which is scored from 0 to 100 with lower scores indicating worse physical function [10]. The second was the Measure Yourself Medical Outcome Profile v2 (MYMOP2), which is a patient generated score. Patients specified their symptoms and limitations for which they were referred to physiotherapy. The follow-up questionnaire was tailored to the patient’s specified symptoms and limitations in order to assess change in those symptoms and limitations [11]. These clinical outcomes were measured at baseline before randomisation and six months later for patients in both the run-in period and main trial.

### Statistical analysis

#### ***Key process measure - telephone call time***

It was envisaged that mean call time would initially be high during the run-in period but would gradually decrease as the physiotherapists grew in confidence and competence in using the new service. Call time was expected to stabilise at around 20 minutes based on the experience of the team responsible for training physiotherapists in the PhysioDirect service [12].

To investigate if, and when, call time had reached a plateau, descriptive statistics compared mean call time between the two phases. The two phases of the trial can be viewed on one timeline as the main trial commenced recruitment immediately after the run-in period thus allowing the call time trend to be explored. The timeline was defined from the date the first patient was referred to physiotherapy and screened for eligibility for the trial in the run-in period to the date that the last patient was referred to physiotherapy in the main trial. Participating PCTs’ timelines were not comparable as each PCT commenced recruitment at different times in the year. Therefore within each PCT, patients were grouped into weeks according to the date of their referral to physiotherapy from the start of the run-in period. A four week rolling mean call time plot illustrated whether there was a trend in call times as patients were recruited to the trial.

Modelling call time trend needs to detect changes in gradient as it was expected physiotherapists’ call times would shorten over time. Joinpoint regression analysis detects these change points (known as a joinpoint) and determines the gradient between joinpoints [13]. Using the timeline described above assumes all physiotherapists had delivered the PhysioDirect service at approximately the same time (that is the start of the run-in period) and had taken the same number of calls per week therefore a different timeline was used. For each physiotherapist, the call times of all their telephone calls were ordered from the earliest date and time of the call. Mean call times were calculated across the physiotherapists in order to represent the mean duration of their first call, second call, third call, and so on. Specifying a maximum of three joinpoints to be used in the modelling, three permutation tests using Monte Carlo methods determined the optimal number of joinpoints needed to describe the mean call time trend: i) testing the null hypothesis of zero joinpoints against the alternative of three joinpoints, ii) testing one joinpoint against three joinpoints, iii) testing two jointpoints against three jointpoints [13]. If no joinpoints were identified, this would suggest the trend had no significant changes of direction; if joinpoints were identified this would suggest there were significant changes in direction which may correspond to when call time had reached a plateau. To determine how long it had taken physiotherapists’ call time to plateau, call times were plotted against the week the patient was referred to physiotherapy, thus identifying when call time had stabilised.

Multilevel models [14] were used to model call time taking into account the clustering effects from the physiotherapists and PCTs; the call times within each physiotherapist were ordered from the earliest date and time of the call. The variance partition coefficient (VPC) was calculated from the null model, which is no adjustment for explanatory variables. This would establish whether the variability in call times was attributable at the PCT level or between physiotherapists within a PCT [15].

#### ***Key clinical outcomes - physical function and symptom change***

To investigate whether patients’ clinical outcomes for those recruited in the run-in period differed to those recruited in the main trial, summary statistics for physical function (SF-36v2 PCS) and symptoms (MYMOP2) at baseline and six months were described for both phases. For each patient, their change in clinical outcome at six months was calculated by subtracting their baseline score from their six month score. A positive change in physical function or a negative change in symptoms indicates improvement in health at six months. Viewing the two phases of the trial on one timeline, a four week rolling mean plot for patients’ change in clinical outcomes at six months showed whether patients recruited in the run-in period had greater or smaller changes in clinical outcomes compared to patients recruited in the main trial.

An analysis of covariance (ANCOVA) compared mean physical function at six months between the PhysioDirect service and usual care (that is the treatment effect) adjusting for baseline physical function, age, gender, PCT and referral problem in the run-in period and the main trial. The clinical effectiveness of the PhysioDirect service and usual care were deemed equivalent if the 95% confidence interval from its treatment effect lay between -2 and 2 points on the physical function scale [9]. An independent *t*-test compared the treatment effect from the run-in period to the main trial [16]. The ANCOVA was repeated for symptoms at six months and replacing baseline physical function with baseline symptoms, followed by the independent *t*-test. For the symptoms scale, a difference of no more than 0.5 points was defined as demonstrating equivalence [17].

Longitudinal modelling [14] was used to model the dynamics of the clinical outcomes at six months for usual care and PhysioDirect over time where time is week of referral to physiotherapy. This would illustrate that patient clinical outcomes did not differ between patients recruited within the same phase or between different phases of the trial. The modelling adjusted for week of patient referral, treatment and phase of the trial plus a three way interaction term.

Joinpoint Regression Programme v4.0.1. [18] was used for the joinpoint regression modelling and all other analyses were conducted in STATA v12 [19].

## Results

### Key process measure - telephone call time

**Figure 1:**
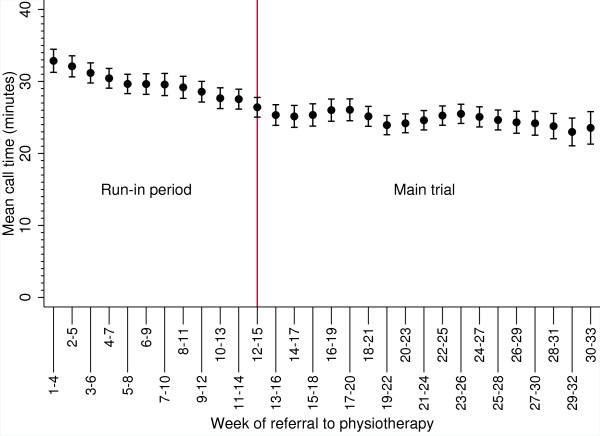
**Call time trend.** Means with 95% confidence intervals.

Thirty-two senior physiotherapists delivered the PhysioDirect service. Mean call time was higher in the run-in period compared to the main trial (31 minutes (SD: 12.6) versus 25 minutes (SD: 11.6)) and the finding was the same when stratified by PCT (Table 1). Figure 1 shows a four week rolling mean plot for call time illustrating call time was initially high at 35 minutes per call during the run-in period but gradually decreased over time and eventually reached a plateau of approximately 25 minutes prior to patient recruitment to the main trial.

**Table 1:** Call time distribution

		**Mean (SD), number of telephone calls**	
		**Run-in period N = 759**	**Main trial N = 1236**
PCT			
	A	31 (12.2), 190	23 (9.3), 395
	B	29 (11.1), 181	27 (9.8), 290
	C	38 (12.7), 255	30 (15.5), 298
	D	23 (7.3), 133	19 (7.5), 253

**Figure 2:**
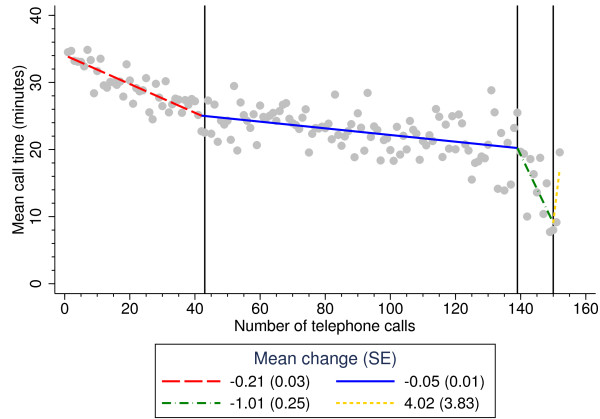
**Identified changes in the call time trend using joinpoint regression.** Between 0 and 41 telephone calls, on average call time decreased by 0.21 minutes (12 seconds) when physiotherapists answered the next telephone call; between 43 and 138 calls, call time decreased by 0.05 minutes (3 seconds); between 140 and 149 calls, call time decreased by 1.01 minutes (60 seconds); between 151 and 152 calls, call time increased by 4 minutes. SE: standard error.

The joinpoint regression model identified three statistically significant changes in the call time trend (Figure 2). The first change occurred at 42 (95% CI: 20, 56) telephone calls hence each physiotherapist, reduced their call time by 0.21 minutes (12 seconds) when they answered their subsequent call, with their call time stabilising when they each had answered 42 calls. It took at least seven weeks from the start of the run-in period for call times to stabilise. The second change occurred at 139 (95% CI: 135, 145) calls and the third at 150 (95% CI: 148, 150); the change in call times in the third and fourth periods may have been associated with a lack of calls made to the service as the trial stopped recruitment to new patients, and the standard error (SE) for the change in call times is wide due to the small number of calls.

From the longitudinal modelling the VPCs were calculated. In total, 30% (95% CI: 25.8, 34.1) of the variation in calls times was attributable at the PCT level which rose to 41% (95% CI: 35.7, 45.8) between physiotherapists within a PCT.

### Key clinical outcomes - physical function and symptom change

There were 1,465 randomised patients in the run-in period and 2,249 patients in the main trial. Patient characteristics (age, gender and referral problem) (Table 2) and clinical outcomes (Table 3) were similar between patients randomised to usual care and PhysioDirect in the run-in period and the main trial.

**Table 2:** Baseline participant characteristics

	**Run-in period**		**Main trial**	
	**Number of patients (%)**		**Number of patients (%)**	
	Usual care	PhysioDirect	Usual care	PhysioDirect
	N = 488	N = 977	N = 743	N = 1506
Age^a^	49.8 (15.9)	50.3 (16.0)	49.0 (16.3)	48.9 (16.0)
Gender:				
Female	288 (59.0)	583 (59.7)	438 (59.0)	897 (59.6)
Male	200 (41.0)	394 (40.3)	305 (41.1)	609 (40.4)
Referral problem:				
Cervical	63 (13.0)	116 (11.9)	89 (12.0)	185 (12.3)
Thoracic	6 (1.2)	19 (1.9)	13 (1.8)	35 (2.3)
Lumbar	139 (28.6)	292 (29.9)	203 (27.4)	412 (27.4)
Upper limb	104 (21.4)	201 (20.6)	174 (23.5)	351 (23.3)
Lower limb	147 (30.3)	302 (30.9)	225 (30.3)	450 (29.9)
Widespread pain	3 (0.6)	5 (0.5)	7 (0.9)	8 (0.5)
Multiple MSK	22 (4.5)	39 (4.0)	27 (3.6)	55 (3.7)
Other MSK	2 (0.4)	3 (0.3)	4 (0.5)	10 (0.7)
PCT:				
A	128 (26.2)	256 (26.2)	251 (33.8)	499 (33.1)
B	118 (24.2)	224 (22.9)	165 (22.2)	348 (23.1)
C	159 (32.6)	342 (35.0)	174 (23.4)	353 (23.4)
D	83 (17.0)	155 (15.9)	153 (20.6)	306 (20.3)

*MSK*: musculoskeletal.

^a^Mean (standard deviation) provided.

**Table 3:** Distribution of clinical outcomes

	**Run-in period**		**Main trial**	
	**Usual care**	**PhysioDirect**	**Usual care**	**PhysioDirect**
Physical function				
Baseline	36.5 (8.9), 486	37.2 (8.9), 975	37.7 (8.6), 743	36.8 (8.9), 1504
6 months	43.7 (10.6), 320	43.9 (10.8), 613	44.2 (10.8), 629	43.5 (10.9), 1283
Symptoms				
Baseline	3.8 (1.0), 485	3.8 (1.0), 975	3.8 (1.0), 743	3.8 (1.0), 1504
6 months	2.2 (1.4), 317	2.3 (1.4), 610	2.4 (1.4), 518	2.4 (1.4), 1033

Mean (standard deviation), number of patients.

**Figure 3:**
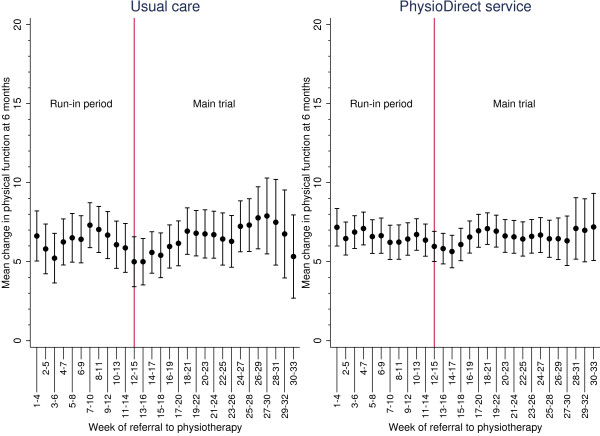
**Patients’ improvement in physical function (SF-36v2 PCS) at six months at different stages of recruitment.** Means with 95% confidence intervals.

**Figure 4:**
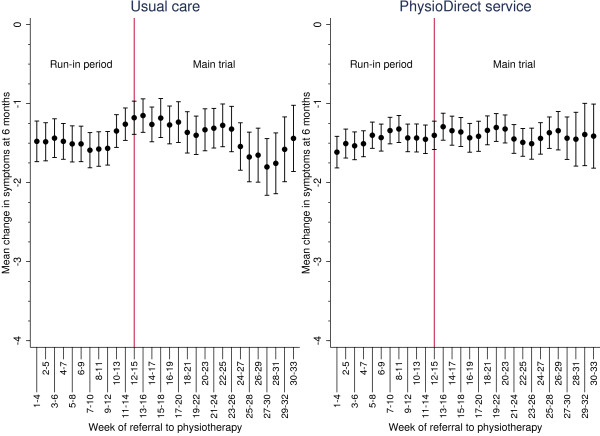
**Patients’ improvement in symptoms using the Measure Yourself Medical Outcome Profile (MYMOP2) at six months at different stages of recruitment.** Means with 95% confidence intervals.

By randomisation arm, Figure 3 and Figure 4 show the rolling mean plot for the clinical outcomes as patients were recruited to the trial. In usual care, although patients’ physical function and symptoms improved, their clinical outcomes varied more at different stages of recruitment compared to the PhysioDirect service where the clinical outcomes were more consistent. This may be associated with the smoothing effect of the larger sample in the PhysioDirect arm.

The difference in patients’ physical function between the PhysioDirect and usual care trial arms at six months was 0.17 (95% CI: -0.91, 1.24) for the run-in period and -0.01 (95% CI: -0.80, 0.79) for the main trial. The 95% confidence intervals lies entirely between -2 and 2 points on the physical function scale suggesting PhysioDirect was as effective as usual care in both the run-in period and the main trial. An independent *t*-test performed between the two treatment effects gave a difference of 0.17 (95% CI: -0.19, 1.53) suggesting there was no significant difference in patients’ physical function achieved during the run-in period and the main trial.

The difference in symptoms for the PhysioDirect service compared to usual care at six months was 0.03 (95% CI: -0.13, 0.20) for the run-in period and -0.02 (95% CI: -0.16, 0.11) for the main trial. The 95% confidence intervals lies entirely between -0.5 and 0.5 points on the symptoms scale suggesting PhysioDirect was as effective as usual care in both the run-in period and the main trial. Performing an independent *t*-test between the two treatment effects gave a difference of 0.06 (95% CI: -0.16, 0.27) again indicating no significant difference in patients’ clinical outcomes from the run-in period and the main trial.

**Figure 5:**
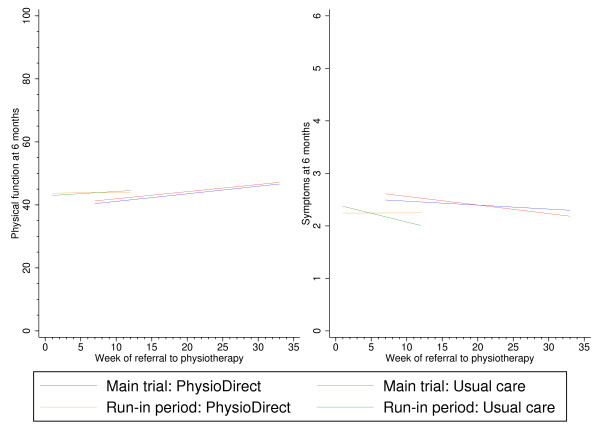
Six month clinical outcomes for PhysioDirect and usual care as patients were recruited to the trial.

Longitudinal modelling of the clinical outcomes in Figure 5 illustrates clinical outcomes at six months of patients recruited in the run-in period did not differ to patients recruited in the main trial nor had patient outcomes differed to those recruited in different weeks within a phase.

## Discussion

### Summary of main findings

This study determined the optimal length of a run-in period of consolidation within a trial testing a new primary care service known as PhysioDirect, and whether the run-in period was necessary before key clinical outcomes were evaluated in the main trial.

Our key process outcome of telephone call time showed that the physiotherapists operating the PhysioDirect service initially required more time to assess and advise musculoskeletal pain patients but that over time their call times reduced. Each physiotherapist needed to answer approximately 42 calls in order for their call time to stabilise at around 25 minutes per patient. A seven week run-in period would have been sufficient for call times to stabilise rather than the longer run-in period used in this trial.

Our results show that the differences in key patient clinical outcomes between the PhysioDirect service and usual care did not change from the run-in period to the main trial. In both phases the new PhysioDirect service was clearly as clinically effective as usual care. Patients randomised to the PhysioDirect arm received the same treatment effects regardless of when they were recruited to the trial. This means that the overall evaluation of the PhysioDirect service could have been based on combining patients from both the run-in period and the main trial to generate more precise estimates.

### Comparison with previous research

Run-in periods are used for a variety of reasons and may be used to select which potential participants can be randomised to RCTs. For example, Ulmer *et al*. [4] conducted an RCT of a behavioural intervention which had a one month run-in period to allow potential participants time prior to randomisation to reflect on whether they would like to continue in the study. As a result the RCT had a lower drop-out rate compared to the trial’s pilot study which did not have a run-in period.

Other studies have investigated the effect of the learning curve on the processes and clinical outcomes in trials. Taekman *et al*. [20] showed that early recruitment was associated with more protocol departures than later recruitment but did not find changes in their key patient outcome (mortality). Laterre *et al*. [21] found greater mortality in participants randomised to the intervention arm who were recruited early on in their ADDRESS and PROWESS trials. Learning curves exist in surgical trials where surgeons improve their performance of a new technique; it has been suggested that the effect of the learning curve can be controlled using post *ad hoc* statistical methods [22].

Although previous research has shown the benefits of run-in periods, no study appears to have investigated the effect of a run-in period of consolidation on key process measures and clinical outcomes when evaluating the effectiveness of a new health service. This may be because previous studies have most likely not included a run-in period, or they have not compared data between the run-in and main trial period, or because the same outcome measures were not collected in both phases.

### Strengths and limitations

Although this study was not powered to detect whether the difference in clinical outcomes between the PhysioDirect service and usual care had changed from the run-in period to the main trial, the PhysioDirect trial had a large number of patients randomised in the run-in period and in the main trial; was conducted in four PCTs and delivered by 32 senior physiotherapists. Considering the size of the recruited samples in the run-in period and the main trial, this may be the largest study to investigate whether key process and patient outcomes change from the run-in period to the main trial and whether a run-in period is necessary.

The fact that the study was conducted over four centres enhances its generalisability. All physiotherapists undertook the same structured training programme and had to be certified as being competent to deliver the PhysioDirect service and they all used the same telephone assessment software to ensure consistency in the way the PhysioDirect service was delivered. However the study did identify some differences in mean call times between the PCTs, which probably reflected other external pressures on the services in each area.

### Implications

The appropriate length of run-in period in a trial testing new health services or treatment approach varies and is dependent on the recruitment rate, randomisation allocation and the training and support that health professionals are given. In a large RCT testing PhysioDirect services, we have demonstrated that a shorter run-in period of seven weeks would have been sufficient for key processes to stabilise. This would have made it possible to recruit and follow up fewer patients.

A learning curve in key processes associated with the delivery of a new service can affect the analysis of its cost-effectiveness. In the PhysioDirect service, physiotherapists initially took longer over patient telephone calls and this would have affected estimates of its running costs in the first few weeks of operation. Conclusions about the cost-effectiveness of the PhysioDirect service compared with usual care should only be based on the main trial because including information from the run-in period would inflate the PhysioDirect’s overall costs and potential lead to incorrect conclusions. In future trials the cost-effectiveness analysis of the new service in its first few weeks of operation would be useful in demonstrating the additional cost from the learning curve.

The run-in period had insufficient power (63%) to detect equivalence between usual care and the PhysioDirect service as its main purpose was to act as a consolidation period to embed the trial’s procedures. In retrospect, patients’ clinical outcomes did not change from the run-in period to the main trial, therefore the two phases could have been combined resulting in a shorter recruitment period of 14 weeks to recruit 625 and 1,250 patients to usual care and PhysioDirect respectively rather than a total of 33 weeks used in the PhysioDirect trial. Future RCTs could build in a run-in period and using similar methods described here, investigate if process measures change over time and determine when learning stabilises. Once determined, the main trial would commence recruiting the remaining participants needed and the final analysis would not acknowledge a portion of the data had come from the run-in period which is an approach taken in an internal pilot study [23]. Future trials could adopt this approach to limit the extra resources needed in conducting a run-in period as a shorter recruitment period would be required.

The PhysioDirect service was set up in four PCTs and they had different lengths of run-in periods. It was decided that each PCT should have a run-in period of at least four to six weeks in order to test out all processes. The first PCT to start the trial used the maximum 12 week run-in period whilst subsequent PCTs required a shorter run-in period as experiences from the first PCT were shared with the other PCTs. Future trials conducted in several services could use this example by allowing the first service team to start the trial to have the longest run-in period and subsequent teams to have shorter run-in periods.

## Conclusions

This study demonstrated that a learning curve existed in the process of delivering a new primary care service known as PhysioDirect. Although a twelve week run-in period of consolidation was allowed for physiotherapists to become competent in delivering the new service, a shorter run-in period of seven weeks would have sufficed for telephone call times to stabilise. Changes in key patient clinical outcomes between PhysioDirect and usual care were the same in the run-in period and in the main trial therefore the PhysioDirect trial did not require a run-in period of consolidation for the purposes of assessing the primary clinical outcome. Future trials should build in a run-in period if it is anticipated learning would have an effect on patient outcomes and use process measures and similar methods presented here to identify when learning stabilises to maximise the efficiency of their trial.

## Abbreviations

ANCOVA: analysis of covariance; MYMOP2: Measure Yourself Medical Outcome Profile v2; PCT: primary care trust; PCS: physical component score; RCT: randomised clinical trial; SD: standard deviation; SE: standard error; VPC: variance partition coefficient.

## Competing interests

The authors declare that they have no competing interests.

## Authors’ contributions

NEF, CS and AAM conceived the study. CS, AAM and NEF undertook acquisition of data. CS, AAM, NEF, JB and TR designed the study. Analysis was undertaken by JB and TR. All authors’ interpreted the data, drafted or revised the article critically for important intellectual content and approved the final manuscript.
